# Spanish validation of Bad Sobernheim Stress Questionnaire (BSSQ (brace).es) for adolescents with braces

**DOI:** 10.1186/1748-7161-5-15

**Published:** 2010-07-15

**Authors:** Elisabetta D'Agata, Carles Pérez Testor, Manuel Rigo

**Affiliations:** 1 Ramon Llull University, Faculty of Psychology. C/Cister, 34, 08022 Barcelona, Spain; 2 E. Salvá Spinal Deformities Rehabilitation Institute, Via Augusta, 185, 08021 Barcelona, Spain

## Abstract

**Background:**

As a result of scientific and medical professionals gaining interest in Stress and Health Related Quality of Life (HRQL), the aim of our research is, thus, to validate into Spanish the German questionnaire **Bad Sobernheim Stress Questionnaire (BSSQ) (mit Korsett)**, for adolescents wearing braces.

**Methods:**

The methodology used adheres to literature on trans-cultural adaptation by doing a translation and a back translation; it involved 35 adolescents, ages ranging between 10 and 16, with Adolescent Idiopathic Scoliosis (AIS) and wearing the same kind of brace (Rigo System Chêneau Brace). The materials used were a socio-demographics data questionnaire, the SRS-22 and the Spanish version of BSSQ(brace).es. The statistical analysis calculated the reliability (test-retest reliability and internal consistency) and the validity (convergent and construct validity) of the BSSQ (brace).es.

**Results:**

BSSQ(brace).es is reliable because of its satisfactory internal consistency (Cronbach's alpha coefficient was 0.809, p < 0.001) and temporal stability (test-retest method with a Pearson correlation coefficient of 0.902 (p < 0.01)).

It demonstrated convergent validity with SRS-22 since the Pearson correlation coefficient was 0.656 (p < 0.01). By undertaking an Exploratory Principal Components Analysis, a latent structure was found based on two Components which explicate the variance at 60.8%.

**Conclusions:**

BSSQ (brace).es is reliable and valid and can be used with Spanish adolescents to assess the stress level caused by the brace.

## Background

The topic of stress has been studied in different situations involving diseases and it is closely related to the HRQOL concept. Stress deteriorates quality of life, which has been defined as a multidimensional concept, and Stress is one of its component [1].

The concept of "stress" has been re-defined and changed over time. At the moment, there are different definitions of stress, mainly divided into three categories:

1. *stress *as a physiological, mainly hormonal, **response **to environmental demands [2];

2. *stress *as a **stimulus **caused by the environment [3];

3. *stress *as a relationship between **the person and the environment**. In this last theory, through a cognitive appraisal, the person has an active role in evaluating situations as exceeding his resources. Lazarus and Folkman (1984) call "coping strategies" the individual's cognitive and behavioral efforts which aim to minimize, reduce, tolerate and dominate environmental demands. According to the authors, there are eight coping strategies and they belong to two groups: Problem solving strategies (such as, Seeking Social Support) and Emotion-focused strategies (such as Escape-Avoidance) [4]

Specifically, since the object of this study is the Adolescent population with Idiopathic Scoliosis (IS), stress response can be roused by spinal deformity and increased by brace treatment [5]. So, bracing is considered a further stressor for adolescents who are just living in a "storm-and-stress" period, characterized by accentuated conflicts with parents, mood disruptions and risk behaviors [6]. Above all in female adolescents, bracing generally correlates with a poor Body Image [7] at a time when it is normally changing and evolving into an adult identity.

Psychological stress is a cause of concern for professionals as it can negatively correlate with no compliance treatment [8,9].

However, researchers reveal that there are some protective factors for treatment outcomes in adolescents with bracing, such as:

- living in an ordinary family rather than living in a single parent family [10];

- having a mother who takes a positive attitude towards her own daughter's scoliosis [11];

- receiving an individual and/or family psychological treatment [8], [12], [13].

Briefly, these are the reasons which have led to assessing HRQOL and stress in brace treated patients. Vasiliadis E, Grivas TB, Gkoltsiou V (2006) and Botens-Helmus C, Klein R, Stephan C (2006) prepared two specific questionnaires for brace treated patients: Brace Questionnaire [14] values the HRQL in adolescents with scoliosis during a brace treatment; Bad Sobernheim Stress Questionnaire (BSSQ (mit Korsett)) measures the Stress produced by brace in adolescent with Idiopathic Scoliosis [15].

The research is aimed at validating the German BSSQ (mit Korsett) into Spanish (Additional File 1), as there are no questionnaires addressing to adolescents with bracing in this language.

## Methods

The design of the study followed the validation process of trans-cultural adaptation. It included two phases: first, a translation and a back translation was used to create the Spanish version, which we called "BSSQ (brace).es"; second, the psychometric characteristics, reliability and validity, were calculated [16].

In 2008, the German version was translated into Spanish by three different professional translators who produced their own version. These were then compared and discussed by an Expert Committee. Another translator rendered the last Spanish version into German and compared the original version with the final one, verifying a satisfactory conceptual equivalence.

The setting was the private centre, E. Salvá, Spinal Deformities Rehabilitation Institute.

In 2008, 35 adolescents took part in this current study. They were all patients with IS wearing the same type of brace (Rigo system Chệneau brace) in part/full time modality, always during the day and outside the home. They agreed to participate in the research as volunteers and their parents signed an informed consent.

Adolescents with non- IS were excluded as well as the ones who wore the brace only at night and not at school.

Specifically, the sample consisted of 35 adolescents: 33 girls and 2 boys, from 10 to 16 years of age with an average age of 13 and a standard deviation of 1.4.

They were all Spanish and 60% of them lived in Catalonia.

**Figure 1:**
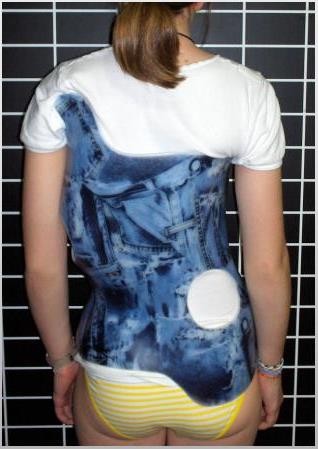
**(Long brace) (Thoraco-lumbar brace with upper thoracic sub axillar extension)**.

**Figure 2:**
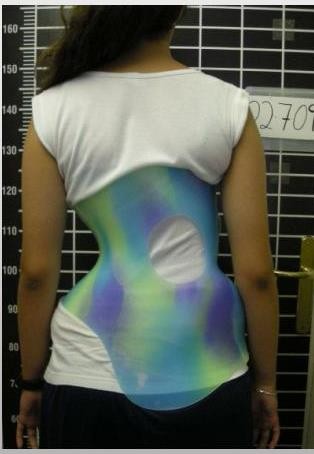
**(Short brace) (Lumbo-sacral brace with a main thoracic counter-pad)**.

Mean Cobb angle of the major curve was 35.42° (SD 11.28°; Range 20°-68°); twenty-one out of 35 patients (60%) wore the brace full-time (23 hours) while 14 (40%) wore it part-time (16 hours, including at school); twenty-six (74.3%) used a 'long brace' design (Figure 1) and 9 patients (25.7%) used a 'short brace' design (Figure 2). Twenty-six patients (74.3%) received physiotherapy at the same time (specific exercises).

From a temporal point of view, adolescents had been wearing the brace for 14 months (SD 21) on average.

The materials used were the following:

• **a Socio-demographic Questionnaire**, created *ad hoc*;

• the **Spanish version of BSSQ (mit Korsett) **[15]. The questionnaire consists of 8 Likert-scale items. A maximum score of 24 indicates the lowest level of stress while 0 means the highest level of stress. The original German version has a high reliability α of 0.97 and a correlation test-retest of 0.88 whereas there is no published study on validity. The questionnaire has not been validated into any other language.

• The **SRS (Scoliosis Research Society)-22 **consists of 22 Likert- scale items associated with 5 domains: function/activity (5 items), pain (5 items), self image- appearance (5 items), mental health (5 items) and satisfaction with the treatment (2 items). Each question presents 5 possible answers which score on a scale from 1 to 5. Higher scores indicate a better patient Quality of Life (QOL).

The BSSQ (brace).es was administered twice to 35 adolescents; the second time occurred after 4-7 days and anonymity was always guaranteed in order to calculate the reliability test-retest.

SPSS.16.0 program was used for the statistical analysis. BSSQ (brace).es mean and standard deviation (SD) were determined as well as the score distribution to determine the floor and ceiling effects (percentage of patients with maximum and minimum score). Shapiro-Wilk test was used for normality and the BSSQ (brace).es score was found to be normally distributed. As a consequence, this allowed the use of parametric analytical processes.

The first statistical property studied was reliability, which means that the questionnaire is trustworthy. The aspects of reliability studied here were "test-retest reliability" and "internal consistency". *Test-retest reliability *studies the capability of the questionnaire to offer constant results over time. It was calculated through the Pearson coefficient which gave a correlation between different scores of the same subject in two different moments. In our research, 3 people did not fill out the second questionnaire. *Internal consistency *analyzes the homogeneity of the questionnaire. It measures the degree of correlation among each item of BSSQ (brace).es, through the Cronbach's α coefficient.

The second statistical property calculated was "validity" to ensure the instrument measures what it is meant to measure. The property calculated in this research was "construct validity", measured by "convergent validity" and "Principal Component Analysis". Convergent Validity was achieved by correlating BSSQ (brace).es scores with those from SRS-22 and its subscales.

SRS-22 mean and SD were calculated as well as means and SD of its subscales.

Shapiro-Wilk test was used to verify normal distribution in our sample according to the values of SRS-22 so that the parametrical analytical process could be used.

An exploratory Principal Component Analysis was used [17], which is a statistical technique that transforms a whole of interrelated items into uncorrelated variables, the Principal Components. "Correlation Matrix" was calculated to find the Pearson correlation coefficients among all items and verify the inter-correlations; Bartlett sphericity test was assessed to prove the lack of interrelations among the items, which is a null hypothesis that was rejected.

From the correlation matrix, a "factor matrix" was extracted, where the Components appeared. Each component was associated to an Eigenvalue, which means the value of variability explicated by the Component (in percentage and in cumulated percentage). To determine the numbers of Components, Kaiser's rule was followed, choosing only the Components whose values is > 1; so that it was possible to identify the two Principal Components: the first Component better explained the total variance while the second Component explained the residual variance.

Factorial matrix was calculated to show the relations between the Component and the items. To simplify its interpretation, a rotated factor matrix was carried out in which each item presented a lower saturation for one factor and a higher saturation for the other one. The rotation Method chosen has been Varimax, the most used, which is applied for non-correlated Factors.

## Results

The result of calculating the average value of BSSQ in the sample was 11.7 (SD 4.8). Floor and ceiling effects were 0% as shown in Table 1, which means that there were no patients with a state of minimum or maximum stress (24-0), according to the Questionnaire. In Table 2 frequency and percentage for each item are shown.

**Table 1:** Frequency and Percentage of BSSQ (brace).es total score

BSSQ (brace).es total score	Frequency	Percentage
3	3	8.6

4	1	2.9

6	2	5.7

7	1	2.9

8	2	5.7

9	2	5.7

10	2	5.7

11	2	5.7

12	4	11.4

13	2	5.7

14	4	11.4

15	2	5.7

16	3	8.6

17	2	5.7

19	1	2.9

20	1	2.9

21	1	2.9

Total	35	100

**Table 2:** Frequency and Percentage of answers for each item in BSSQ (brace).es

Items	Answers to the Items	Frequency	Percentage
**Item 1**	Strongly agree	**9**	**25,7**
	
	Agree	**20**	**57,1**
	
**I feel uncomfortable by the appearance of my body in the brace**	Disagree	**5**	**14,3**
	
	Strongly disagree	**1**	**2,9**

**Item 2**	Strongly agree	**7**	**20,0**
	
	Agree	**13**	**37,1**
	
**It is hard for me to be open with my brace.**	Disagree	**7**	**20,0**
	
	Strongly disagree	**8**	**22,9**

**Item 3**	Strongly agree	**9**	**25,7**
	
	Agree	**11**	**31,4**
	
	Disagree	**10**	**28,6**
	
**I feel uncomfortable in situations where other people can see my brace.**	Strongly disagree	**5**	**14,3**

**Item 4**	Strongly disagree	**6**	**17,1**
	
	Disagree	**9**	**25,7**
	
**I don't feel embarrassed when people see my brace**	Agree	**14**	**40,0**
	
	Strongly agree	**6**	**17,1**

**Item 5**	Strongly agree	**9**	**25,7**
	
	Agree	**12**	**34,3**
	
**I avoid body contact so that no-one knows that I wear a brace**.	Disagree	**13**	**37,1**
	
	Strongly disagree	**1**	**2,9**

**Item 6**	Strongly agree	**15**	**42,9**
	
	Agree	**12**	**34,3**
	
**When deciding what kind of clothes to wear or how to wear my hair, I try to make sure my brace is hidden**	Disagree	**5**	**14,3**
	
	Strongly disagree	**3**	**8,6**

**Item 7**	Disagree	**3**	**8,6**
	
	Agree	**11**	**31,4**
	
**I don't feel embarrassed to show my brace to people close to me (parents, friends and school- friends)**	Strongly agree	**21**	**60,0**

**Item 8**	Strongly agree	**4**	**11,4**
	
	Agree	**8**	**22,9**
	
**Because of my brace I avoid activities/hobbies, which otherwise I love to do**.	Disagree	**12**	**34,3**
	
	Strongly disagree	**11**	**31,4**

The BSSQ (brace).es distribution in relation to age was normal in our sample as Shapiro-Wilk test verified.

The test-retest reliability was found to have an excellent high temporal stability (Pearson correlation coefficient 0.9, p < 0.01 for a sample of 32). Moreover, BSSQ (brace).es had a good internal consistency because the Cronbach's α value was significant (0.8) for a sample n = 35.

Besides, the convergent validity was studied between BSSQ (brace).es and SRS-22 as well as between BSSQ (brace).es and SRS-22 subscales. Normal distribution in our sample was verified according to the values of SRS-22 so that the parametrical analytical process could be used.

SRS-22 had an average value of 89.7 (SD 10), while mean Function/Activity Subscale was 21.4 (SD 2.5), mean Pain subscale was 22.4 (SD 3.2), mean Self Image subscale was 16.9 (SD 2.9), mean Mental Health subscale was 20.1 (SD 3.2) and mean Satisfaction subscale was 8.9 (SD 1.4), as it is shown in Table 3.

**Table 3:** Descriptive data of SRS-22 and its subscales

	N	Minimum	Maximum	Mean	Std. Deviation
SRS-22	35	63	105	89.7	10

Function/Activity	35	16	25	21.4	2.5

Pain	35	11	25	22.4	3.2

Self-Image	35	11	24	16.9	2.9

Mental Health	35	13	25	20.1	3.2

Satisfaction	35	6	10	8.9	1.4

The general correlation between BSSQ (brace).es and SRS-22 was satisfactory (r = 0.656, p < 0.001), as it was expected; Pearson correlation coefficient between BSSQ (brace).es and SRS-22 scales were: Function 0.43 (p = 0.05), Pain subscale 0.37 (p = 0.05), Self Image 0.60 (p = 0.01), Health Mental 0.61 (p = 0.01) and Satisfaction 0.45 (p = 0.05). Pearson Correlations are shown in Table 4.

**Table 4:** Correlation matrix among BSSQ and SRS-22 subscales

SRS-22 subscales	function/Activity	Pain	Self Image	Mental Health	Satisfaction
**BSSQ(brace).es**	0.43*	.3 7*	.60*	.61*	.45*

*. Correlation is significant at the 0.05 level (2-tailed). ** Correlation is significant at the 0.01 level (2-tailed).

Moreover, in BSSQ (brace).es two Principal Components were found. In *Correlation Matrix *there were low Pearson correlations between item set 8 and 7 with item set 1 and 2; it assessed low correlations between these pairs of items. In *Bartlett sphericity test*, the null hypothesis was not verified (χ^2 ^= 88.8, df = 28, p < 0.001) because correlations existed among the items.

Through a *Factor Matrix*, two Components explained the 60.8% of cumulative variance: Factor 1 explained 43.8% of the total variance and Factor 2 explained 17% of the rest (Table 5). Table 6 represents the *Factorial Matrix *for the two Components. Table 7 corresponds to the Factorial Matrix after rotation (Varimax type), showing the component each item belongs to.

**Table 5:** Factor Matrix for the Principal Component Analysis.

	Initial Eigenvalues		
	
Components	Total	% of Variance	%Cumulative Variance
1	3.5	43.8	43.8

2	1.4	17	60.8

3	.9	10.6	71.4

4	.7	8.5	79.9

5	.6	7.5	87.4

6	.5	5.8	93.3

7	.3	4	97.3

8	.2	2.7	100

Initial values of variance explicated by the Components (Eigenvalues).

**Table 6:** Components Matrix

Component	1	2
Item 1	.6	-.4

Item 2	.7	-.4

Item 3	.8	-.1

Item 4	.7	.3

Item 5	.7	-.4

Item 6	.6	.1

Item 7	.5	.6

Item 8	.5	.6

**Table 7:** Rotated Components Matrix (Varimax Rotation Method)

Component	1	2
Item 1	.7	.0

Item 2	.8	.1

Item 3	.7	.4

Item 4	.4	.7

Item 5	.8	.1

Item 6	.4	.5

Item 7	.0	.8

Item 8	.0	.7

In Table 7, it was clear that items 1 and 2 belong to Component 1, and items 7 and 8 belong to Component 2, while the other items (4,5,6,3) were in between the two factors (items 3, 5 belong to Component 1 and items 4, 6 to Component 2).

This is shown below (the items translated above are not validated in English [15]):

Factor 1:

Item 1.I feel uncomfortable by the appearance of my body in the brace

**Item 2.It is hard for me to be open with my brace**.

**Item 3.I feel uncomfortable in situations where other people can see my brace**.

**Item 5.I avoid body contact so that no-one knows that I wear a brace**.

Factor 2:

Item 4.I don't feel embarrassed when people see my brace

Item 6.When deciding what kind of clothes to wear or how to wear my hair, I try to make sure my brace is hidden

Item 7.I don't feel embarrassed to show my brace to people close to me (parents, friends and school- friends)

Item 8. Because of my brace I avoid activities/hobbies, which otherwise I love to do

## Discussion

As shown in Table 8, the Spanish sample (n = 35) was smaller than the German one (n = 62) and it was more homogeneous because it was composed of adolescents from the same centre and with the same type of brace. Therefore, future research hopes to increase the sample size to include adolescents wearing different types of braces. Besides, probably due to sample size, ceiling and floor effects are 0%; it would be advisable to check them in a larger sample. Above all, a statistical analysis with a larger sample has to be assessed for item n. 7 - I don't feel embarrassed to show my brace to people close to me (parents, friends and school- friends) -as nobody in our sample chose the alternative "strongly disagree". It is probably due to the fact that showing the brace to people close to the adolescent is not considered a very stressful situation. Probably this item could be changed in the future.

**Table 8:** Synthetic table of comparing validation process of the German original version (BSSQ (mit Korsett) with the trans-cultural Spanish adaptation.

	BSSQ (mit Korsett)	BSSQ (brace).es
Sample size	n = 62	n = 35

BSSQ Mean score	12.5	11.7

Reliability. Test-retest	r = 0.88	r = 0.90

Reliability. internal consistency	α = 0.97	α = 0.81

Convergent validity	-	r = 0.66

The average value of BSSQ in the sample was a mean value of stress, considering 0 the highest value of stress and 24 the lowest. Furthermore, test-retest reliability and internal consistency had values that were as high as in the German research (Table 8).

Unlike in the German study, construct validity was calculated. To assess convergent validity, SRS-22 was used because, as literature shows, Stress and Quality of Life are correlated. Moreover, it is validated in Spanish and has good statistical properties. The general correlation was satisfactory because higher stress correlates with lower Quality of Life; more specifically, high scores in BSSQ (low stress) correlate with high scores in SRS-22. Consequently, the highest correlations between BSSQ (brace).es and SRS-22 subscales were related to Self Image and Mental Health. Therefore, that result indicated a relationship between the concepts of Stress and Body Image, and Stress and Mental Health; thus stress roused by brace was co-related to the appearance and connected to suffering in adolescents, as literature confirms.

In the exploratory Principal Component Analysis, how the items were related to the components was not totally clear, possibly because the sample size and item numbers were reduced and because the questionnaire presented a high homogeneity trait.

It could be possible to increase the amount of items in order to introduce new Components related, such as, Family Relationship or Social Support, as they have strong influences on the adolescent's life.

So, the two Components could be interpreted as one bipolar (Stress/No stress), since stress is the result of a personal cognitive evaluation (Lazarus, 1996). In that manner, Factor 1 could be identified with the *Presence of Stress *or "**Embarrassed feelings in public situations" **such as wearing braces could be uncomfortable (item 1, 3), hard (item 2) in social situations or the adolescent could avoid body contact (item 5); while, Factor 2 could be considered as *Lack of Stress *or "**Brace adaptation"**: patients do not feel embarrassed when people see their braces (item 4, 7), they avoid favorite activities (item 8) or pay close attention to their choice of clothes or how to wear their hair (item 6). In Factor 1 and 2, we found the use of coping strategies (Lazarus and Folkman, 1984), above all Escape-Avoidance (item 5, 6 and 8).

## Conclusions

▪ The questionnaire seems to be a useful instrument to measure stress in adolescents during brace treatment for spinal deformity. The statistical features analyzed were satisfactory.

▪ However, the small amount of items has limited the validation process, and future research should continue analyzing its structure in order to clarify the Components. Nevertheless, the strong point of the instrument is its brevity, since it can be easily used by doctors and rehabilitators as an everyday clinical tool.

▪ The main importance of the present work lies in it being the first Spanish questionnaire to refer to Stress for adolescents wearing braces.

## Consent

Written informed consent was obtained from the patients and their relatives for publication of this article. A copy of the written consent is available for review by the Editor-in-Chief of this journal.

## Competing interests

Grant "Fundació Jesús Serra y Universidad Ramon Llull" was given to DE for PhD 2008 and Ortholutions funding was received for the German- Spanish translations.

All other authors declare that they have no competing interests.

## Authors' contributions

All the authors participated in every phase of the study and read and approved the final manuscript. Specifically, DE participated in its design, in its data collection, performed the statistical analysis and drafted the manuscript. PTC directed the work from a methodological point, participated in its design and coordination, helped in drafting the manuscript. RM conceived the study, participated in its design and coordination, helped in drafting the manuscript

## Authors' information

▪ **DE**: PhD Student in Clinical Psychology at Ramon Llull University, Barcelona;

▪ **PTC**: Doctorate in Medicine and Surgery, specialist in Psychiatry. Professor in Psychopathology and Psychology and Family at Psychology Faculty, Ramon Llull University, Barcelona. Director of the Psychological Medical Centre and Coordinator of the Couple and Family Unity in Vidal i Barraquer Foundation.

▪ **RM**: Doctorate in Medicine and Surgery; Specialist in Spinal Deformities Rehabilitation at Elena Salvá Institute, Barcelona. Lecturer at the Physiotherapy School "Gimbernat", Universidad Autónoma, Barcelona.

## Supplementary Material

Additional file 1 **BSSQ (brace).es (The Spanish validated version)**. Click here for file
