# Supplementary material for: Spanish validation of Bad Sobernheim Stress Questionnaire (BSSQ (brace).es) for adolescents with braces
Source: Scoliosis. 2010 Jul 15;5:15. doi: 10.1186/1748-7161-5-15 (PMC2913914; doi:10.1186/1748-7161-5-15)
Supplement: Additional file 1 — BSSQ (brace).es (The Spanish validated version). [file 1748-7161-5-15-S1.PDF]

GRPF
grup de recerca
de parella i família
Universitat Ramon Llull

Facultat de Psicologia, Ciències
de l'Educació i de l'Esport Blanquerna
Universitat Ramon Llull

Cuestionario sobre Escoliosis y Estrés

BBSQ (brace).es[1]

Las siguientes preguntas se refieren a cómo te sientes con el corsé, es decir cuando llevas el corsé puesto. Rellena, por favor, el cuestionario con atención y sinceridad. El análisis de los cuestionarios nos permitirá evaluar mejor las incomodidades provocadas por el corsé e introducir mejoras para su futuro tratamiento.

Señala tu grado de acuerdo con las frases siguientes

1. **Me afecta la apariencia de mi cuerpo con el corsé puesto.**
   - ☐ Muy de acuerdo
   - ☐ De acuerdo
   - ☐ En desacuerdo
   - ☐ Muy en desacuerdo

2. **Me cuesta mostrarme en público llevando el corsé.**
   - ☐ Muy de acuerdo
   - ☐ De acuerdo
   - ☐ En desacuerdo
   - ☐ Muy en desacuerdo

3. **Me resultan desagradables situaciones en las que otros pueden ver mi corsé**.
   - ☐ Muy de acuerdo
   - ☐ De acuerdo
   - ☐ En desacuerdo
   - ☐ Muy en desacuerdo

[1] Traducido y validado del alemán por Alomar, E.; Castillo J. A.; D'Agata E.; Pérez Testor C.; y Rigo M. (2008), con permiso de Weiss, R. y cols., (Botens-Helmus, C., Klein, R. y Stephan, C. (2006). The reliability of the Bad Sobernheim Stress Questionnaire (BSSQ brace) in adolescents with scoliosis during brace treatment. *Scoliosis, 1* (22).

4. **No me afecta enseñar mi corsé.**
   - ☐ Muy de acuerdo
   - ☐ De acuerdo
   - ☐ En desacuerdo
   - ☐ Muy en desacuerdo

5. **Evito el contacto físico para que otras personas no se den cuenta de que llevo corsé.**
   - ☐ Muy de acuerdo
   - ☐ De acuerdo
   - ☐ En desacuerdo
   - ☐ Muy en desacuerdo

6. **Al escoger mi ropa o llevando el cabello largo, procuro esconder mi corsé**.
   - ☐ Muy de acuerdo
   - ☐ De acuerdo
   - ☐ En desacuerdo
   - ☐ Muy en desacuerdo

7. **No me importa mostrar mi corsé a personas cercanas (padres, amigos, compañeros de estudios).**
   - ☐ Muy de acuerdo
   - ☐ De acuerdo
   - ☐ En desacuerdo
   - ☐ Muy en desacuerdo

8. **Debido al corsé renuncio a actividades de ocio y aficiones que me agradan.**
   - ☐ Muy de acuerdo
   - ☐ De acuerdo
   - ☐ En desacuerdo
   - ☐ Muy en desacuerdo

Muchas gracias por tu colaboración.
